# Supplementary material for: Are carbon emissions trading and green financial instruments synergistic? -Comprehensive quantitative research based on content analysis
Source: PLoS One. 2024 Mar 7;19(3):e0298601. doi: 10.1371/journal.pone.0298601 (PMC10919601; doi:10.1371/journal.pone.0298601)
Supplement: S1 Data — (ZIP) [file pone.0298601.s001.zip › Data/地级市Z_F工作报告.zip!/╔╜╬≈/┴┘╖┌╩╨/└┤╫╘╣1⁄2╓┌║┼í╛┬φ┐╦╩2╛▌═°í┐_2014.docx]

各位代表：

现在，我代表市政府向大会报告工作，请予审议，并请市政协委员和其他列席人员提出意见。

一、2013年工作回顾

过去的一年，在省委、省政府和市委的正确领导下，在市人大、市政协的监督支持下，市政府团结带领全市人民，全面贯彻落实党的十八大、十八届二中、三中全会和习近平总书记系列重要讲话精神，扎实推进“稳增长、调结构、促改革、惠民生”等重点工作，圆满完成了各项任务。全市地区生产总值完成1223.6亿元，同比增长8.5%；规模以上工业增加值完成661.8亿元，增长12.6%；公共财政预算收入完成118.2亿元，增长6.7%；城镇居民人均可支配收入完成21936元，增长10.3%；农民人均纯收入完成7768元，增长12.6%。

一年来，我们主要做了以下几方面的工作：

一是积极应对下行压力，经济保持平稳健康发展。

全力扶持实体经济。认真落实中央、省宏观调控政策，推出“煤炭25条”、“强化经济运行20条”、支持中小微企业发展等政策“组合拳”。开展“煤矿基本建设年”活动，新增投产矿井20座，新增产能2340万吨，全市原煤产量达到7041万吨。先后组织全市煤炭、煤焦、煤电、银企和重点项目供需对接会，千方百计帮助企业解决生产经营困难，促进了经济的平稳运行。

强化投资支撑。扎实开展“项目推进年”活动，实施省市重点项目394项，全年项目储备完成20888亿元，项目签约完成3019亿元，项目落地完成1705亿元，项目开工完成1407亿元，省市县重点工程建设完成1157亿元，项目投产完成1250亿元。在项目工作12项全省排名中，我市夺得2个第二、3个第三。

全社会固定资产投资突破千亿元大关，完成1036.3亿元，增长26%。

加快市场建设。社区便民商圈、蔬菜直销点、“万村千乡”、新网工程等城乡流通体系建设成效明显，锦悦城、华纳新天地、居然之家等投入运营，奥特莱斯、红星美凯龙、生龙国际、上东世纪等有序推进。社会消费品零售总额完成475.5亿元，增长13.7%。

二是着力推进转型综改，产业结构调整的步伐明显加快。

综改区建设扎实推进。制定实施了转型综改区建设《三年实施方案》和《2013行动计划》，全面铺开产业转型、生态修复、城乡统筹、民生改善四大任务，扎实推进“五规合一”、户籍制度、用地制度、金融创新和医药卫生体制等五项重大改革，确定了10个重大项目，完成投资58亿元。省级试点侯马市、市级试点尧都区在“村改居”、金融、土地、区域协同发展机制等方面取得了积极进展。

经济带建设取得实效。坚持规划指导、基础先行、产业入园、两区同建，加快建设“百里汾河新型经济带”。《经济带发展战略规划》编制完成；滨河东路南北延路基工程基本完成，河道水利工程洪洞至襄汾段基本完工，生态绿化完成1700多万平方米；18个工业园区销售收入占到全市规模以上企业销售收入的63.9%，10个农业园区有8个形成规模，6个物流园区有4个开工建设，5个文化旅游园区全部实施开发，11个“两区同建”工程启动实施。经过全市人民的共同努力，特别是沿汾各县（市、区）的艰苦奋斗，百里汾河新型经济带可以说已经初具雏形，展现出强劲的发展活力和良好的发展前景，正在成为临汾转型跨越发展的强大引擎。

产业结构不断优化。工业加快转型提升，焦化行业45户企业完成省定兼并重组任务，钢铁行业完成立恒整合中宇工作，淘汰落后炼铁产能75万吨、炼钢产能100万吨。实施新装备、新技术、新材料、新能源等转型项目104个，全市高新技术企业发展到23家，省级以上企业技术中心达到19家，新兴产业投资增速达到50.6%。农业获得稳步发展，“大水网”涉临工程进展顺利，累计完成投资2.81亿元。全年完成灌溉面积214万亩，水土流失治理41.6万亩，生态修复80万亩。粮食总产达到23.2亿公斤，再创历史新高。“四个百万亩”基地和现代农业园区建设规模进一步扩大，新发展设施蔬菜4.4万亩、水果14.2万亩、干果19.8万亩、中药材11万亩。大力推广“公司+合作社+农户”，新确立“一村一品”专业村232个，新发展农民专业合作社1513个，家庭农场达到1348个，农业产业化龙头企业达到362家。三产呈现蓬勃兴起，物流业规模持续扩大，山西国际陆港园区建设加快推进，正在积极申报临汾综合保税区。旅游业稳步发展，共接待国内外游客2143万人，旅游总收入达到195亿元，4A级景区达到6个。金融、保险等服务业迅猛发展。三产所占比重提高1.82个百分点。

三是统筹推进城乡建设，城乡一体化进程全面提速。

交通基础设施建设步伐加快。全市公路通车总里程达到18025公里，同比新增209公里，全省排名第一。霍永高速东段和西段一期工程基本完工，吉河高速路基和桥涵完成60%，大运高速土门连接线拓宽改造工程全面完成。霍侯一级路北段改造和桃临线霍州至汾西段竣工投用。新建改建农村公路231公里，其中集中连片特困地区农村公路36公里。

临汾机场航站楼和高架桥主体完工。大西高铁、中南铁路、张台铁路加快建设。

城市建设加快发展。完成河西新城和空港园区控制性详细规划，完成临汾高铁站地下空间利用等规划，市区控规覆盖率达到85%；建成市规划展览馆、市行政服务监察中心、中大街中段贯通等工程；启动实施了五一东路、二中路、北外环拓宽改造、秦蜀路南延、市民广场一期、集中供热九期、污水处理厂改扩建、市区天然气置换等工程；城市管理得到加强，多年困扰市区交通秩序的三轮车得到有效整治。县城建设取得显著成绩，城市功能进一步健全，控规覆盖率达到70%。

新农村建设取得新进展。30个新农村连片示范区、300个重点推进村建设任务全面完成；农村“五件实事”超额完成年度任务，行政村街道亮化全面完成。

生态环境明显改善。先后出台绿色生态工程、创建园林城市、PM2.5防控、市区大气污染防治等实施方案，狠抓节能减排、大气污染防治、水环境修复治理和农村生态保护，实施节能改造项目76个，万元工业增加值能耗下降3.5%，六项主要污染物减排超额完成省定任务。按照新的《环境空气质量标准》评价，市区二级以上天数达到167天，其中一级18天，空气质量指数全省排名第三，PM2.5达标率好于省定标准。汾河水质得到明显改善，化学需氧量和氨氮分别下降37.93%和20.93%。大力实施“两山”“两网”“两林”“两区”和“双保”林业五大工程，新造林62.6万亩，超省定任务19个百分点。涝洰河河道治理工程全面启动，完成投资1.87亿元。

四是夯实基础落实责任，发展环境更加和谐稳定。

集中教育整顿活动成效明显。认真开展“夯实基础管理、落实工作责任”集中教育整顿活动，围绕安全生产、社会稳定、民生改善等方面，共查找整改安全隐患和突出问题1.5万个，建立制度15452项，构建了分级负责、层层落实的制度体系和责任体系。

安全生产形势稳定好转。开展“安全生产稳定年”活动，强化两个主体责任，推进“隐患排查、专项整治和打非治违”三项行动，不断加大企业安全标准化建设力度，实施ABCD分级分类动态监管，加强对煤矿、非煤矿山、尾矿库、危险化学品、森林防火、冶金、食品药品、道路交通、地质灾害等重点行业和领域的安全监管，关闭取缔非法企业439家。全年生产经营性事故同比下降132起，没有发生重特大事故，全市安全生产形势持续稳定好转。

社会治理持续加强。全市信访工作连续两年实现“四下降一好转”。全面推广安泽县“311”基层服务管理、乡宁县“三化一卡”、侯马市“345”流动人口管理、铁路安全“线格化”管理等先进经验，积极创新社会治理。深入推进“平安临汾”创建，健全社会治安防控体系，依法打击违法犯罪活动，妥善应对和有效处置各类突发事件，社会保持和谐稳定。

五是尽心竭力改善民生，人民群众得到更多实惠。

社会事业全面进步。市图书馆、博物馆等工程加快实施。市直学校改造工程全部投用，5个县薄弱校改造全面完成，全市新建、改扩建农村学校169所、公办标准化幼儿园30所、村级幼儿园65所。临汾新医院建筑工程竣工，正在进行设备安装，市精神病医院主体完工，市第三人民医院迁建工程进展顺利，市第四人民医院和市妇幼保健院成功创建“三甲”，完成9家县级公立医院改革，新农合参合率达99.12%。市区成功创建省级卫生城，16个县被评为省级以上卫生县城。人口自然增长率、免费孕前优生健康检查、出生人口性别比综合治理三项计划生育指标圆满完成，被省政府评为综合先进市。

扶贫开发扎实推进。实施“百企千村产业扶贫工程”，启动7个项目，总投资17.4亿元。与陕西省榆林市合作，实施吕梁山有机苹果产业开发项目，在全国率先实现产业扶贫项目跨省协作。认真开展易地扶贫搬迁、住村联户、片区开发、整村推进等工作，实现贫困人口脱贫6.1万人。

社会保障水平不断提高。全年新增城镇就业6.3万人，城镇登记失业率为2.84%；各项社会保险累计参保人数达到468.2万人；开工建设各类保障性住房26333套，完成农村危房改造8000套。

双拥优抚、国防动员和民兵预备役工作扎实开展，外事、侨务、人防、统计、气象、地震、地方志、档案、老龄、残联、工商联、红十字会、妇女儿童等工作均取得新成绩。

市政府自觉接受市人大的法律监督、工作监督和市政协的民主监督，政府系统承办的人大代表建议和政协委员提案全部办结。依法行政和“六五”普法工作扎实开展，廉政建设、行政监察、审计监督和纠风工作进一步加强。大幅度精简行政审批事项，提高办事效率，市本级保留行政审批事项112项，缩减75%，平均审批时限由3周压缩为1周。严格执行“八项规定”和“约法三章”，清理违规公务用车331辆，停止新建楼堂馆所23个，“三公经费”支出比上年下降15.8%。

各位代表，过去的一年，在严峻复杂的形势下，我们克服各种困难，全市经济社会实现了持续稳定健康发展，成绩来之不易，弥足珍贵。这是省委、省政府和市委正确领导的结果，是市人大、市政协和社会各界监督支持的结果，是全市人民团结一心、奋力拼搏的结果。在此，我代表市政府，向全市广大工人、农民、知识分子和各级干部，向驻临部队、武警官兵和公安干警，向各民主党派、工商联、人民团体和社会各界，表示崇高的敬意和衷心的感谢！在总结成绩的同时，我们也清醒地看到，我市发展还存在一些突出矛盾和问题。

主要是：发展不足、发展不优、发展不平衡的矛盾仍然较为突出,转型升级任务艰巨；科技创新能力不强；县域经济总量偏小，扶贫开发任重道远；安全、环保压力依然较大；民生事业欠账较多；思想解放的程度、改革创新的力度与形势任务的要求尚有差距,政府职能转变、效能提升和工作落实仍存在薄弱环节,“四风”问题还不同程度存在，等等。对于这些困难和问题，我们一定高度重视，采取有力措施，切实加以解决。

二、2014年主要任务2014年是贯彻落实十八届三中全会精神、全面深化改革的重要一年。总体上看，我市仍处于可以大有作为的黄金发展期，中部崛起、转型综改区建设等政策叠加效应更加凸显，深化改革推动发展的空间更加广阔。同时也要看到，全国经济进入增长速度换挡期、结构调整阵痛期交织阶段，我市发展面临的内外部环境更加复杂多变，经济下行压力加大，前进道路上还有更多的挑战和困难。对此，我们既要做好应对困难局面的准备，又要坚定发展信心，牢固树立战略思维和底线思维，全力推动转型跨越发展。

今年政府工作的总体思路是：高举中国特色社会主义伟大旗帜，全面贯彻党的十八大、十八届二中、三中全会和十二届全国人大二次会议精神，贯彻落实习近平总书记系列重要讲话精神，深入开展党的群众路线教育实践活动，认真落实省委、省政府和市委的决策部署，坚持稳中求进的总基调，深入开展改革创新年、转型攻坚年、环境提升年、服务群众年活动，着力推动发展，着力深化改革，着力改善环境，着力保障民生，加快建设文明开放、富裕和谐的新临汾。

2014年全市经济社会发展的主要预期目标是：GDP增长9%，财政总收入增长9%,公共财政预算收入增长6%，规模以上工业增加值增长13%，全社会固定资产投资增长21%，社会消费品零售总额增长14%，城镇居民人均可支配收入增长10%，农民人均纯收入增长11%，城镇登记失业率控制在4.2%以内，居民消费品价格水平涨幅控制在3.5%左右，万元GDP综合能耗下降3.5%，万元GDP二氧化碳排放量下降3.7%，万元工业增加值用水量下降5.5%，二氧化硫、化学需氧量、氨氮、氮氧化物、烟尘、粉尘的减排完成国家和省下达的任务。

实现上述目标，必须坚持解放思想开阔眼界，创造性地贯彻落实上级决策部署；必须坚持大胆改革激发活力，着力破除各种体制机制障碍；必须坚持创新驱动加快转型，推动经济发展提质增效；必须坚持改善民生服务群众，让发展成果惠及全市人民。我们要重点抓好以下八个方面工作：（一）全面深化改革。

大力推进转型综改。以转型综改试验区建设为统领，实施好三年综改试验方案和2014年行动计划，落实“16112”重大改革任务，力争在重点领域改革方面取得突破。

深化国有企业改革，进一步建立现代企业制度，鼓励发展混合所有制经济，开展非国有资本参与国企改革试点工作。深化财税体制改革，完善预算管理制度，建立健全政府性债务管理制度和风险预警机制，积极推进煤炭资源税从价计征。深化投资体制改革，创新金融产品，扩大股权、债权融资。

深化开发区建设机制改革，探索实施临汾、侯马经济开发区扩区工作，大力发展“飞地经济”，在园区发展、基础设施建设以及利益分享等方面积极探索创新。深化招商引资工作机制改革，创新招商引资模式，变项目招商为要素招商、环境招商。深化农村土地制度改革，认真做好土地承包经营权确权登记颁证试点工作，加强土地经营权流转管理和服务，推动土地经营权等农村产权流转交易，探索建立城乡统一的建设用地市场，开展农村集体经营性建设用地出让、租赁、入股试点工作。深化林权制度配套改革，规范林权登记管理，完善林权地籍信息管理系统。按照中央和省的要求,分类推进其他领域改革，全面完成好各项改革任务。

加快建设“百里汾河新型经济带”。将经济带纳入晋陕豫“黄河金三角”地区合作发展规划，力争上升为国家区域发展战略。

编制出台产业发展、生态建设、园区推进、城镇化等方面的专项规划。建立统一运作、利益分享的新机制，对经济带的规划、建设、布局和资源配置实行统一领导，加快推进基础设施建设和产业园区发展。临汾一级客运西站投入使用；滨河东路贯通工程和滨河西路北延工程完工通车；完善临汾—襄汾、临汾—洪洞城际公交。18个新型工业园区、10个现代农业园区、6个物流园区和5个文化旅游园区要健全服务保障体系，完善园区功能，不断提升项目承载能力、就业吸纳能力和人口容纳能力。打造“百里汾河文化旅游创意长廊”，建设汾河生态国家级体育公园。

实施创新驱动战略。制定《国家创新驱动发展战略临汾行动计划（2014-2020年）》和《临汾市低碳创新行动计划》。创新科技发展机制，加大对企业产品开发、品牌创建、市场开拓的支持力度,积极申报省级、国家级科技项目，构建产学研相结合的技术创新体系。促进科技和产业融合发展，围绕煤焦铁等传统产业升级改造、新兴产业做大做强和农业“四个百万亩”基地建设，重点布局一批科技攻关项目，培育一批创新型企业和高新技术企业。创新人才引进和培育机制，加强“两高”人才和技术人才队伍建设，建立博士后流动站、院士工作站，大力引进各类高层次、高技能人才。全市科技进步贡献率达到50%以上，专利申请量增长10%以上。

各位代表，改革是最大的红利，我们要进一步破除体制机制束缚,激活发展要素,努力推动改革创新，让改革发展的成果更多地惠及全市人民！（二）促进经济平稳健康发展。

强化经济运行调节。着力研究和解决经济运行中的突出矛盾和问题，促进经济平稳健康发展。建立重点企业PMI、主导产品市场价格指数等监测平台，强化经济运行监测预警。细化扶持骨干企业和中小微企业发展的政策措施，积极组织电力运力协调会、银企洽谈会，加强对煤、电、油、运、资金等生产要素的协调配置，保障企业正常运转。清理规范涉企行政事业性收费项目，减轻企业负担。强化产销衔接，积极开拓市场，加大对困难行业和企业的帮扶力度。支持煤炭、焦化、钢铁等上下游企业开展多种形式的联合协作，增强企业抵御市场风险的能力。

进一步扩大消费。加快发展现代商贸服务业，抓好奥特莱斯、红星美凯龙、生龙国际、上东世纪等重大商贸项目建设。深化提升“万村千乡”市场、社区蔬菜直销点、放心早餐工程，加快建设城市商贸中心、特色商业街、“15分钟便民商圈”和农产品现代流通体系，发展一批农超对接、农校对接、农餐对接企业。严厉打击制售假冒伪劣产品、价格欺诈、计量欺骗等不法行为，让人民群众安全、放心消费。

努力促进投资增长。扎实开展“项目见效年”活动，按照“六位一体”的要求，全面加大项目投资、协调、服务力度，进一步增强全市经济发展活力和发展后劲。全年项目储备20000亿元，签约2000亿元，落地1700亿元，开工1300亿元，建设1300亿元，投产1200亿元，实施省市重点建设项目383项，总投资6333亿元，今年完成投资916亿元。

激发民营经济活力。进一步加大支持非公经济发展力度，全力推进小微企业创办、“小升规”企业成长、“小巨人”企业培育工程。积极搭建中小企业服务平台，新建小微企业服务站25个。按照“非禁即入、非限即许”原则，研究制定民间投资负面清单，支持民间资本以独资、参股、控股等多种方式进入基础设施、市政公用设施和教育、文化、医疗卫生等领域。年销售收入超亿元“小巨人”企业达到11户，新办中小微企业2000户，创建国家和省级中小企业创业示范基地5个。

（三）加快产业转型升级步伐。

改造提升传统产业。坚持“以煤为基、多元发展”，改造提升煤、焦、冶、电“四大”传统产业。加快推进煤矿“四矿四化”建设，全市煤矿安全质量标准化一级矿井达到10座，其他矿井达到三级以上标准，新增投产矿井20座，原煤产量力争达到8000万吨。继续推进焦化行业兼并重组，淘汰落后焦化产能370万吨，延伸煤化工下游链条。推动区域钢铁企业联合重组，支持组建晋南钢铁集团。提升钢铁企业装备水平，加快产品升级换代，淘汰落后钢产能260万吨、生铁产能33万吨。促进电力工业高效清洁发展，利用好省政府出台的“低热值煤发电20条”，积极推进低热值煤发电项目的申报和核准。

培育壮大新兴产业。加快发展装备制造、新能源、新型材料、电子信息等产业，以煤炭机械、汽车零部件、工程机械配件、家电配件为发展重点，不断提高装备制造业产品生产工艺水平和市场占有率，稳步向总成和整机装配延伸，重点抓好新兴际华高端铸件、铃木电梯、平阳重工等项目。着重发展风电、煤层气、光伏等新能源产业，重点加快梅亿新能源汽车产业园、浮山华润风电、永和煤层气开发利用、侯马志盛150兆瓦太阳能电池组件等项目。承接沿海电子产业转移，建设电子信息产业集群，重点抓好新百佳数码、惠建电子、志盛新能源等电子类龙头企业。加快侯马开发区国家电子商务示范基地、易商中国电子商务平台、“同城购”购物平台等项目建设，推进电子商务快速发展。大力发展新型建材和高性能结构材料等新型材料，重点抓好鸿鼎10万吨新型分离材料、晋盛微晶石、山西普泰发泡铝二期等项目。

提升现代服务业水平。大力发展文化、旅游、物流、金融等产业，带动服务业发展提速、水平提升、比重提高，全市服务业增加值占比提高一个百分点以上。按照产业化运作的模式，整合重组优势资源，推进文化创意、文化传播、文化产品制作、文化交流娱乐等产业发展，重点加强剪纸、刺绣、锣鼓等非物质文化遗产的保护、传承和开发。围绕创建“中国优秀旅游目的地城市”，推进大槐树、壶口等国家5A级旅游景区和汾河公园、东岳庙、彭真纪念馆等4A级旅游景区创建工作，实施晋国都城大遗址保护与核心地带规划开发，晋国博物馆建成投用。充分发挥区位优势，将物流业发展与城市建设有机结合，重点推进山西国际陆港、临汾空港经济区等现代物流园区建设，努力把临汾打造成为全国性的现代物流枢纽城市。重点支持金融业发展，充分发挥驻临金融机构服务经济社会发展的作用，积极引进更多商业银行、保险公司、信托担保机构落户临汾；大力发展金融租赁、投资组合、商业代理、保险中介、消费信贷等金融业务，增强金融服务功能；进一步深化农村信用社改革，促进农村信用社向商业银行转变；加强对担保公司、贷款公司的监管；促进保险业快速健康发展。积极发展育幼养老、家政服务、健康休闲等服务业。

（四）进一步做好“三农”工作。

发展特色现代农业。坚持“一村一品”、“一县一业”的主攻方向，走特色、高质、高端、高效的发展路子。推进“四个百万亩”特色农业基地和8个“一县一业”基地县建设，全年新发展水果5万亩，设施蔬菜3万亩，干果10万亩，中药材5万亩，配套建设100万头生猪基地，新增233个“一村一品”专业村。推进粮食高产创建工程，推广机械化保护性耕作等先进农机技术，增强农业综合生产能力，搞好粮食收储，确保粮食安全，全年粮食总产稳定在20亿公斤以上。大力推进畜禽标准化养殖，稳步提升畜禽综合生产能力，全年肉、蛋、奶的产量分别达到24万吨、14万吨和6.8万吨。高水平打造浮山“印象田园”、翼城菁铧、曲沃太子滩、洪洞秦壁等现代农业园区。抓好“两平台一通道”建设，巩固完善市、县、乡、村农业科技服务体系。继续实施农业产业化“393”工程，重点培育年销售收入超亿元以上龙头企业15家，力争全年农产品加工企业销售收入突破70亿元。

提高农村公共服务水平。实施农村人居环境改善工程，加快农村路、水、电、气等基础设施建设，逐步改善农村生产生活条件。加快新农村建设，做好农村学校、农村文化体育设施和服务标准化工作。继续办好“五件实事”。大力实施乡村清洁、农村困难家庭危房改造、农村幼儿园改造、贫困人口易地搬迁工程，培训1万名新型职业农民。

扎实搞好扶贫开发。加大扶贫开发力度，实施精准扶贫，完成贫困人口脱贫6万人，易地扶贫搬迁7150人，完成整村推进39个村，确保贫困地区农民人均纯收入增幅高于全市平均水平。全面推进“百企千村”产业扶贫开发工程，加强与阳煤、焦煤、晋煤、国新能源4大省属企业的对接协作，加快实施国新能源6000吨酸枣深加工、晋煤集团恒温库、阳煤集团食用菌等项目；引导市内企业参与扶贫开发，与贫困村进行“一对一”帮扶，加快项目对接和资金落实，今年在每个县（市、区）打造1—2个标杆企业。

实施“千村万人”就业培训计划，确保年内5000名贫困劳动力通过培训实现就业。深入推进连片特困地区扶贫攻坚，加快推进吕梁山集中连片特困地区山地有机苹果产业化项目，实施干鲜果畜禽循环项目。开展领导干部包村增收、机关定点扶贫工作。继续实施古县、安泽等片区开发项目。

各位代表，“三农”工作事关全局，扶贫攻坚责任重大。我们一定带着对农民群众的深厚感情，切实抓好“三农”工作，扎实推进扶贫开发，让农业更强，让农村更美，让农民更富！（五）积极推进新型城镇化。

加快建设中心城市。建立和完善城市规划体系，中心城区控规覆盖率达到100%。按照“拓展、改造、提质”的总体思路，着力打造汾河两岸核心区，建设河西新城和东部新区，逐步疏散老城区，实现城市品质总体提升。河西新城突出完善交通网络，拉大城市框架，年内完成滨河西路北延、规划三街南段、新高中南路等城市道路工程，加快实施漪汾花园南路、锣鼓桥西延、规划三街中段等城市道路工程，力争年内建成通车，初步形成连接南北、贯通东西的新路网。加快推进市图书馆、博物馆、美术馆、奥体中心、市民广场等公共基础设施建设，全面提高城市承载能力。老城区着力畅通交通网，年内完成五一东路贯通、二中路拓宽改造、北外环东段拓宽改造、秦蜀路南延等工程，加快推进中大街贯通、迎春南街拓宽改造、西赵路等路网工程。改造城中村2个、城郊村18个。

完善新型城镇体系。坚持遵循规律、因势利导、顺势而为、水到渠成的指导思想，坚持以人为本、优化布局、生态文明、传承文化的基本原则，完善和实施“一带两圈多点”的城镇化规划，努力提升城镇化质量和水平。加快百里汾河新型城镇带、临汾都市圈和侯马都市圈、12个大县城和60个重点镇建设。按照“三个1亿人”的战略部署，促进农业转移人口落户城镇，引导农村人口就近城镇化。积极推进户籍制度改革，逐步放宽落户条件，进一步完善就业、养老、上学、医疗等各项配套政策，引导农村居民向城镇集聚。加快城市棚户区、国有工矿棚户区、林区棚户区改造步伐，今年改造各类棚户区6400户。

加快城乡交通基础设施建设。坚持高速公路、国省干线、农村公路、旅游公路“四网并重”，公铁空立体交叉，同步发展。全市在建和开建高速公路331公里，完成129公里，完成集中连片特困地区农村公路195公里。霍永高速东段和西段一期工程8月底实现通车，吉河高速年底完成路基工程，霍永高速西段二期争取年内开工。积极支持大西高铁、中南铁路建设，完成大西高铁客运站及站前广场、景观大道等工程，大西高铁7月份正式通车。加快临汾机场建设，力争年底试航，规划建设机场连接滨河东路、108国道、长临高速3条快速通道。

（六）加快推进生态文明建设。

狠抓大气污染防治。围绕涉气企业、煤烟污染、尾气排放和扬尘治理四个重点，认真落实我市大气污染防治实施方案和行动方案。从严治理涉气企业污染，加快市区和县城周边钢铁、焦化、建材企业搬迁步伐；加快推进集中供热、供气改造，加强餐饮企业油烟治理，重点实施市区天然气置换和26个“城中村”、80个“城郊村”的燃煤炉灶气化改造；加强机动车尾气治理，坚决淘汰“黄标车”；强化扬尘治理，严格建筑工地、企业周边及道路运输扬尘监管，加大街道除尘保洁力度。建立大气污染源清单，加强PM2.5监测预报，提高重污染天气的应对能力，确保我市大气污染防治工作走在全省的前列。

深入推进水环境治理。实施主要河流地表水跨界断面考核，全面启动实施北城壕沟排水、向阳路防汛、污水一厂扩容、河西污水处理、龙祠水源净化五大环保工程，市区和17个县城实现管网铺设到位、污水收集处理达标到位、垃圾收集处理到位，确保河流水质持续改善。

强力推进节能减排。积极申报国家节能减排财政政策综合示范城市。进一步加大节能评估、能效对标和节能执法力度，开展千家企业节能低碳行动，重点实施50个节能改造项目。持续推进“十城万盏”半导体照明应用示范工程。大力推进结构减排、工程减排和管理减排，严格控制新上高耗能、高污染项目，建设完善企业减排监控系统，确保污染减排监控数据传输率达到75%以上。

保护和修复生态环境。进一步加大“两山”“两网”“两林”“两区”和“双保”林业五大工程建设力度，重点实施高速公路通道绿化、吕梁山生态脆弱区植被恢复两个标杆项目，完成营造林45万亩。创建省级园林城市，启动国家园林城市创建工作，重点实施街道绿化工程，继续加快涝洰河生态环境综合治理等生态工程。做好国家主体功能区建设试点工作，制订西山片区试点实施方案，推动形成符合生态文明要求的生产方式和生活方式。结合草地确权，大力开展天然牧坡改良和人工种草，完成牧坡改良20万亩，人工种草20万亩。

各位代表，推进生态文明建设，是民之所想、民之所盼，我们要坚定不移地走生态优先的路子，让临汾的天更蓝、水更清、空气更洁净！（七）着力推进社会建设。

繁荣发展文化事业。大力实施“文化强市”战略。加快市县乡村文化基础设施建设，提升公共文化服务水平。以“大美临汾”为主题，开展“群众文化基层行”活动。加强对外文化交流，扩大临汾对外影响。加快广播电视发展步伐，加强对新兴数字媒体的监管。扎实开展“扫黄打非”工作。广泛开展全民健身活动。

持续改善教育、医疗条件。坚持办好人民满意的教育，深化教育综合改革，全面实施素质教育，提升教育质量；加快发展学前教育，均衡发展义务教育,推进义务教育阶段学校标准化、信息化建设；全面提升高中教育发展水平，大力发展职业教育,积极发展特殊教育、成人教育，规范发展民办教育；积极推进山西师大和临汾学院新校区建设。优化配置医疗资源，以临汾新医院搬迁为契机，启动市区医疗资源整合工作，打造以“三甲”医院为龙头的医疗联合体；建成市精神病医院，加快市第三人民医院建设；巩固新型农村合作医疗制度，确保参合率稳定在95%以上；完善国家基本药物制度，全面深化县级公立医院改革，大力发展中医药事业。坚持计划生育基本国策不动摇，启动实施一方是独生子女的夫妇可生育两个孩子政策，全市人口自然增长率控制在6.5‰以内。

全面加强社会保障。坚持保基本、兜底线、促公平、可持续，抓好各类社会保险提标扩面工作。推进城乡居民保险制度整合和管理服务一体化，提高失业、工伤、生育保险待遇水平，做好医疗保险城镇居民门诊统筹试点工作，实行社会保障“一卡通”。

不断完善城乡低保、大病救助、住房保障等制度，新开工建设各类保障性住房18500套，基本建成13200套。进一步扩大住房公积金覆盖面。做好弱势群体和困难群众的救助工作。

持续推进社会治理创新。健全落实重大决策社会稳定风险评估机制,加强和改进信访工作，认真开展“百日双百案”群众信访诉求化解专项活动，完善矛盾纠纷排查调处机制，及时就近化解社会矛盾，加强基层社会服务体系建设，健全村务公开、居务公开和民主管理制度，更好发挥社会组织在公共服务和社会治理中的作用。进一步完善网络舆情监管机制。加强社会治安综合治理，扎实推进“平安临汾”、“法治临汾”建设，严厉打击各类违法犯罪活动，提高人民群众的安全感。加强应急救援管理，提高公共安全和防灾救灾减灾能力。深化全民普法教育，做好法律援助，加强社区矫正和安置帮教工作。

（八）切实抓好安全生产。

我们要把安全生产放在经济社会发展全局的重要位置，牢固树立“红线”意识、责任意识和担当意识，坚持“三个决不能过高估计”的基本判断，严格执行安全生产法律法规，全面落实安全生产责任，完善安全生产长效机制。开展“知责、履责”活动，健全完善安全生产岗位责任制，明确安全生产职责，形成知责制度化、履责规范化的整体氛围。深入实施企业ABCD分级分类动态监管，建立科学监管长效机制。按照“全覆盖、零容忍、严执法、重实效”的要求，深入开展“打非治违”活动，坚决打击任何名目、任何形式的私挖滥采行为。持久开展安全生产大检查，切实抓好煤矿、非煤矿山、危险化学品、尾矿库、水库、道路交通、桥涵隧道、地质灾害、森林防火、食品药品、特种设备、建筑施工、学校等重点行业和领域的安全隐患排查治理。加大安全投入，强化人员培训，推进科技兴安。加快消防基础设施、消防装备和消防队伍建设。全面构建市县乡村四级食品药品安全监管体系，启动建设市食品药品检验检测中心。严格落实安全生产目标责任考核“一票否决制”，严格事故责任追究，努力减少一般事故，有效防范较大事故，坚决杜绝重特大事故，为改革发展提供安全保障。

在做好上述工作的同时，我们还要深入开展“双拥共建”活动，做好国防动员、人民防空、民兵预备役工作，促进军民融合式发展。支持工会、共青团、妇联等人民团体更好地开展工作。依法推进第十届村民委员会换届选举。搞好第三次全国经济普查和第一次全国地理国情普查。统筹做好民族宗教、外事侨务、对台、老龄和残疾人等工作，提升住房公积金、统计、气象、水文、档案、地方志和防震减灾等工作水平。

各位代表，民生问题始终是政府的工作重点。为群众办实事，政府责无旁贷。今年，我们要结合开展党的群众路线教育实践活动，实施“六大惠民工程”。

实施学前教育优质普惠工程。启动实施第二轮学前教育三年行动计划，做好民办及城镇小区配套幼儿园清理整顿工作，完善城镇小区配套幼儿园规划建设与使用管理办法，抓好新建小区配建幼儿园工作，努力增加公办幼儿园数量，完成新建、改扩建30所标准化公办幼儿园和改造33所农村幼儿园的任务，实现学前三年毛入园率达75％以上，提高学前教育优质普惠水平。

实施乡村医疗服务提质工程。进一步提高新农合保障水平，人均筹资标准由340元提高到390元，政策范围内住院费报销比例稳定在75%左右。完善落实基本药物制度。深入推进乡村卫生服务一体化管理，全面落实乡村医生多渠道补偿政策，稳定乡村医疗队伍。

实施农业强基工程。加强农田水利基础设施建设，结合全省“大水网”工程布局，搞好我市“小水网”配套工程，重点抓好中部引黄、禹门口东扩、引沁入汾浮山供水等工程建设，完成洪洞曲亭水库修复工程，完成11座中小型病险水库治理任务，不断提高防洪灌溉能力。扎实推进高标准基本农田建设，大力实施中低产田改造。投资1.55亿元，实施规模化节水、膜下滴灌、田面灌溉项目，逐步解决农民浇地“最后一公里”问题。创新农业耕种模式，提高农机装备和作业水平，推广机械化保护性耕作和农机深松整地技术。落实种粮直补、农资综合直补等各项惠农政策。

实施饮水安全工程。加强水源地保护、供水管网改造、水质检测等工作，让全市人民喝上安全水、放心水。全面排查整治水源地环境安全隐患，实施龙祠等饮用水源周边环境综合整治。市区新建城市供水网8.3公里，改建3.5公里，各县市新建城市供水网47.6公里，改建29.5公里。改善和提高5万农村人口饮水安全标准，加强农村饮用水检测，提高全市农村自来水入户率，落实农村供水工程水价补贴政策。

实施城市综合整治工程。突出治堵、治乱、治脏“三大环节”，全面推进城市管理上台阶。在治堵上，从公交优先、停车位建设等方面着手，多管齐下，最大限度缓解交通压力。优化城市公共交通结构，实现高铁车站、民航机场、城西一级客运站、火车站等重要交通节点的公交车、出租车的有效衔接。建立公安交警和城管执法联动机制，对车辆违章和违法占道经营等实施集中整治，规范城市交通秩序。在治乱上，深入开展门店招牌、户外广告、临街建筑门面等专项整治活动，建立长效管控机制。在治脏上，以主次干道、集贸市场、车站和城乡结合部为重点，严格落实沿街单位门前“五包”责任制，对临街污染大、脏乱突出的行业进行集中整顿，对城乡结合部垃圾堆放等问题进行彻底整治，认真做好垃圾收集、清运和处理工作。加快建设以数字化、网络化、智能化为主要特征的智慧城市，提升城市信息化管理水平。推广洪洞“六城联创”先进经验，加强县城城市管理，扮靓市容市貌，创造一个整洁、优美、舒适的工作生活环境。

实施就业创业援助工程。把就业放在更加突出的位置，制定出台就业扶持和就业援助实施办法，组织实施高校毕业生创业引领计划、“三支一扶”计划、就业见习计划，建设市、县、乡、村四级联网的城乡就业人才服务网络体系，开展就业咨询、信息发布、职业介绍等服务，举办不同形式、不同类型的人才招聘和交流活动，切实促进城乡就业。加大财政投入，有计划地开发购买公益性岗位，帮助零就业家庭人员、“4050”人员等就业困难人员实现就业。启动就业培训全覆盖计划，全年培训城镇失业人员8800人，农村劳动力23500人，新成长劳动力6000人，创业培训3000人，今年新增城镇就业岗位51000个。

各位代表，改善民生是政府一切工作的出发点和落脚点，我们就是要从群众最关心、最迫切的事情做起，把关系民生的工作一项一项抓紧抓好、一件一件落到实处，让人民群众得到更多的实惠，感受到更多的温暖和幸福！各位代表，打铁还需自身硬，完成今年各项发展任务，必须切实加强政府自身建设。我们一定按照“为民、务实、清廉”的要求，振奋精神，落实责任，努力建设法治政府、创新政府、务实政府、高效政府和廉洁政府，不断提高工作能力和服务水平。

建设法治政府。自觉接受人大及其常委会的法律监督与工作监督,自觉接受政协的民主监督，自觉接受社会公众和舆论监督。广泛听取民主党派、工商联、无党派人士意见，充分发挥工会、共青团、妇联等人民团体作用。支持法院、检察院依法独立公正行使职权。严格按照法定权限和程序行使权力、履行职责。坚持科学决策、民主决策、依法决策,加强调查研究,完善重大行政决策咨询论证和公众征询制度。

建设创新政府。坚持解放思想，深化改革，创新行政管理理念和方式，健全决策、执行、监督机制，大胆破除制约科学发展的各种障碍，推进精细化管理，不断提高工作决策指导能力和解决实际问题能力。

加快政府机构改革步伐，年内完成工商、质监行政管理体制调整和卫生与人口计生机构整合。加快事业单位分类改革，推进行业协会、商会与行政机关脱钩。加快政府向社会购买服务的改革，建立健全政府购买公共服务机制。

建设务实政府。扎实开展第二批党的群众路线教育实践活动,大力学习弘扬焦裕禄精神，自觉践行“三严三实”要求，着力解决“四风”突出问题，深入基层,深入一线,推动作风建设常态化、长效化。深入推行“一线工作法”,确保工作任务不折不扣落到实处。对主要指标、重点工作和重大项目实行目标责任制考核，强化目标刚性约束,真正以目标引领行动、用考核推动落实。切实改进文风会风,精简会议和文件,严格控制节庆、论坛、展会等活动。

建设高效政府。扎实推进简政放权，深化行政审批制度改革，在承接好中央、省里下放的审批事项的同时，要继续精简审批事项，市级审批事项在112项的基础上，再缩减10%。积极开展行政事业性收费项目专项整治，清理取消不合法、不合理的收费项目。充分发挥市行政服务监察中心作用，搭建行政审批、财政资金使用、公共资源交易等社会公共服务与纪律监察为一体的网络平台，健全完善“联合审批、限时办结、超时默许”工作机制，行政审批全部进大厅，所有公共资源交易一律阳光运行，简化办事程序，优化审批流程，缩短审批时限，推行“一站式”服务，一个窗口对外，面向社会公开，接受群众监督，方便群众办事，优化发展环境。设立市长信箱和市长热线电话，畅通群众诉求渠道。

建设廉洁政府。严格执行中央“八项规定”、国务院“约法三章”和省、市有关要求，落实好“一岗双责”,完善权力制衡机制,真正把权力关进制度的笼子。认真执行廉政准则,强化效能监察和审计监督。严格落实《市直机关会议费管理办法》，对会议费实行综合定额控制。严控“三公”经费支出和楼堂馆所建设,强化“三项治理”，把艰苦奋斗、厉行节约的要求,落实到政府运行各个环节。深入推进阳光政务,扩大政府信息公开范围。

各位代表，新的航程已经开启，前进的号角催人奋进。让我们紧密团结在以习近平同志为总书记的党中央周围，在市委的坚强领导下，团结和带领全市人民，攻坚克难，锐意进取，为率先转型，全力跨越，加快建设文明开放、富裕和谐的新临汾而努力奋斗！
